# Introduction to *Case Reports in Pancreatic Cancer*

**DOI:** 10.1089/crpc.2015.29005.cjy

**Published:** 2015-11-01

**Authors:** Charles J. Yeo

**Affiliations:** Department of Surgery, Thomas Jefferson University, Philadelphia, Pennsylvania.

I would like to welcome the readers of this new publication—*Case Reports in Pancreatic Cancer*. It is critical at this point in history that we focus our attention on pancreatic cancer.

Pancreatic cancer is currently the fourth leading cause of cancer deaths in the United States; however, it is projected to rise in both numbers and rankings. A recent executive summary from the Pancreatic Cancer Action Network (PanCAN) predicts that pancreatic cancer will move from the fourth leading cause of cancer deaths in the United States to become the second leading cause of cancer deaths in the United States by perhaps 2020 or 2025. Importantly, it is notable that pancreatic cancer is alone among the top five U.S. cancer killers (currently lung, colorectal, breast, pancreas, and prostrate in that order) in that the incidence rate and the death rate are increasing only for pancreatic cancer. Shockingly, but important to repeat, pancreatic cancer is poised to be the number two cause of cancer death in the United States in the next decade.

Many of us have been privileged to work in the field of pancreatic cancer for almost three decades. Progress has been made. However, sadly there currently are no universally applicable early detection tools or “major breakthroughs” that have arisen in the field of pancreatic cancer. The pancreatic cancer research community is small, although growing, and has had some successes. The pancreatic cancer research community has sadly been underfunded by the National Cancer Institute (NCI).

Fortunately, the U.S. Congress has passed the Recalcitrant Cancer Research Act, which has asked the NCI to formulate a comprehensive and long-term strategic plan to bring the infrastructure, intellectual energy, and resources into play so that we can more efficiently attack this urgent cancer diagnosis.

I was honored to have been asked to serve as the editor-in-chief of this new journal in the field of pancreatic cancer. There are many journals that publish on pancreatic cancer: journals in the cancer field, surgical field, medical oncology field, pathology field, and so on. However, informative case reports can be difficult to publish, although each of them can serve an important educational mission. We have put together an outstanding group of associate editors, from our Jefferson Pancreas and Biliary Related Cancer Center. I am extremely pleased to welcome the following individuals who will join me as associate editors:

- Voichita Bar-Ad, MD
- Jonathan R. Brody, PhD
- Thomas Kowalski, MD
- Harish Lavu, MD
- Ashwin R. Sama, MD
- Jordan M. Winter, MD
- Theresa P. Yeo, PhD

In the oncoming year, we will be rapidly developing the journal into an international publication by expanding the editorial board and adding international associate editors. Moreover, we are excited to receive and publish case reports or case series that deal with various topics in pancreatic cancer from all over the world. These may vary from demographics and epidemiology of pancreatic cancer, discussions of various inherited pancreatic cancer syndromes, pathological findings, molecular genetics, interesting radiographical findings, surgical treatment, anatomical abnormalities, odd complications of surgery or chemotherapy, medical oncology observations, radiation oncology observations, and so on. We would love to receive manuscripts that deal with not only surgical interventions but also endoscopic interventions, and endoscopic diagnoses, endoscopic palliation, interventional radiology techniques, neoadjuvant treatment strategies, postresection adjuvant therapy, as well as supportive modalities in the palliative setting. We are also interested in novel therapies that may include immunotherapy, monoclonal antibodies, or other unusual or cutting-edge therapy.

We are excited to proceed with this new journal venture. My thanks go to the publisher, Mary Ann Liebert, Inc., who has made this dream into a reality.

## Abbreviations Used

NCI: National Cancer Institute
PanCAN: Pancreatic Cancer Action Network

